# Expression of Kin17 promotes the proliferation of hepatocellular carcinoma cells *in vitro* and *in vivo*

**DOI:** 10.3892/ol.2014.2244

**Published:** 2014-06-12

**Authors:** WEI-ZHENG KOU, SU-LING XU, YING WANG, LI-WEI WANG, LEI WANG, XIAO-YAN CHAI, QIN-LIANG HUA

**Affiliations:** 1 Department of Oncology, First Affiliated Hospital of Xinxiang Medical University, Weihui, Henan 453100, P.R. China; 2 Department of Ultrasound, First Affiliated Hospital of Xinxiang Medical University, Weihui, Henan 453100, P.R. China; 3 Department of Laboratory, First Affiliated Hospital of Xinxiang Medical University, Weihui, Henan 453100, P.R. China; 4 Department of Cardiology, First Affiliated Hospital of Xinxiang Medical University, Weihui, Henan 453100, P.R. China

**Keywords:** Kin17, hepatocellular carcinoma, proliferation

## Abstract

Kin17 protein is ubiquitously expressed in mammals and is correlated with vital biological functions. However, little is known about the role of Kin17 in the proliferation of hepatocellular carcinoma cells. The aim of the present study was to investigate whether the upregulation of Kin17 can promote the growth of hepatocellular carcinoma cells. A series of assays was performed to study the effect of Kin17 in the proliferation of hepatocellular carcinoma cells *in vitro* and *in vivo*. The western blotting results revealed that Kin17 expression was increased in hepatocellular carcinoma tissues compared with that of the corresponding normal tissues. Moreover, ectopic upregulation of Kin17 expression promoted the growth of hepatocellular carcinoma cells *in vitro* and *in vivo*. These results indicated that Kin17 is involved in the tumorigenesis of hepatocellular carcinoma, and that Kin17 has the potential to serve as a therapeutic target for hepatocellular carcinoma.

## Introduction

Hepatocellular carcinoma is a common malignancy and is the third leading cause of cancer-related mortality worldwide. Although great breakthroughs have been achieved in the diagnosis and treatment of hepatocellular carcinoma, recurrence and metastasis of the disease continue to affect the prognosis of patients. Therefore, in order to improve the prognosis of hepatocellular carcinoma patients, there is an urgent requirement for the development of new therapeutic targets (1).

Human Kin17 is a 45-kDa nuclear protein that is remarkably conserved during evolution (2–4). The Kin17 protein is ubiquitously expressed in mammals and is correlated with vital biological functions (5). There exists a region of 40 residues in the core of Kin17, which is homologous to RecA protein. Kin17 protein possesses three motifs: A zinc finger, a bipartite nuclear localization signal and the core domain that is homologous to RecA protein. To date, the following major roles of Kin17 have been identified: i) binding to curved DNA and recombination of human cells (6,7); ii) complementing the functions of H-NS and controlling gene expression (8); and iii) upregulation by UVC or ionizing radiation (9–11). Thereby, Kin17 is associated with mRNA processing, involving gene transcription and cell cycle regulation. Taking into account the uncontrolled growth and unlimited replication potential of hepatocellular carcinoma cells, Kin17 may therefore be involved in the pathogenesis and progression of hepatocellular carcinoma.

In the present study, the expression of Kin17 was investigated in hepatocellular carcinoma tissues and hepatocellular carcinoma cell lines. A series of experimental methods *in vitro* and *in vivo* were used to explore the possible role of Kin17 in the carcinogenesis of hepatocellular carcinoma. To the best our knowledge, this is the first study to evaluate the effects of Kin17 on the growth of hepatocellular carcinoma.

## Materials and methods

### Cell lines and tissues

Hepatocellular carcinoma cell lines, HepG2, SMMC-7721 and BEL-7402 (American Type Culture Collection, Manassa, VA, USA), were maintained in RPMI-1640 (Gibco-BRL, Carlsbad, CA, USA) medium containing 10% fetal bovine serum (Hangzhou Sijiqing Biological Engineering Materials Co., Ltd., Hangzhou, China), 100 μg/ml penicillin and 100 μg/ml streptomycin (both Invitrogen Life Technologies, Carlsbad, CA, USA). Hepatocellular carcinoma cells were transfected with the full-length human Kin17 cDNA or a control pcDNA3.0 vector (both Yinru Biological Engineering., Ltd., Changsha, China) using Lipofectamine 2000 (Invitrogen Life Technologies). The colonies were selected with G418 (550 μg/ml) for 3 weeks and expanded. HepG2 and SMMC-7721 cells with overexpression of Kin17 were classified as the HepG2-Kin17 and SMMC-7721-Kin17 groups, respectively. Similarly, the equivalent pcDNA3.0-transfected cells were termed as the HepG2-Vector and SMMC-7721-Vector groups, respectively. Eight fresh, paired hepatocellular carcinoma and noncancerous tissue samples were used for the western blot analysis, along with the hepatocellular carcinoma cell lines. The specimens were obtained from patients at the Department of Oncology, First Affiliated Hospital of Xinxiang Medical University (Weihui, China). Informed consent was provided by each patient, and the study was approved by the ethics committee of the First Affiliated Hospital of Xinxiang Medical University.

### Western blot analysis

After dicing the paired cancerous and normal samples, these tissues were extracted in cell lysis buffer (radioimmunoprecipitation assay; Thermo Fisher Scientific, Waltham, MA, USA) with protease inhibitors. A total of 1×10^6^ HepG2, SMMC-7721 and BEL-7402 cells were washed twice with ice-cold phosphate-buffered saline (Fuzhou Maixin Biotechnology Development Co., Ltd., Fuzhou, China) and lysed with cell lysis buffer at 4°C for 30 min. The lysates were acquired by centrifugation at 20,000 × g for 15 min at 4°C. Equal amounts of proteins from HepG2, SMMC-7721 and BEL-7402 cells or paired cancerous and normal tissue samples were boiled for 8 min prior to being loaded onto 10% polyacrylamide gels (Kangwei Century Co., Ltd., Beijing, China) and transferred to polyvinylidene fluoride membranes (Millipore, Billerica, MA, USA). Following incubation with the monoclonal mouse anti-human Kin17 antibody (1:150 dilution; Santa Cruz Biotechnology, Inc., Santa Cruz, CA, USA), the horseradish peroxidase-conjugated secondary polyclonal goat anti-mouse IgG (1:4500 dilution; Beijing Zhongshan Golden Bridge Biotechnology Co., Beijing, China) was incubated for 2 h at room temperature. Finally, Kin17 was visualized by enhanced chemiluminescence (Electro-Chemi-Luminescence system; Beijing Shengke Ruida Technology Development Co., Ltd., Beijing, China). The membranes were then re-blotted with anti-GAPDH antibody for normalization and confirmation of equal protein loading. Due to the vital roles of cyclin D1 and p27Kip1 in regulating cell proliferation, the effect of Kin17 on their expression was detected. CyclinD1 (polyclonal mouse anti-human cyclinD1 antibody, 1:100 dilution, Santa Cruz Biotechnology, Inc.) and p27Kip1 (monoclonal mouse anti-human p27Kip1 antibody, 1:200 dilution, Santa Cruz Biotechnology, Inc.) expressions were studied according to the method described previously. Bandscan 5.0 software (Glyko, Novato, CA, USA) was used to analyze the results of the western blot analysis. Compared with the BEL-7402 cells, the endogenous expression of Kin17 was lower in the HepG2 and SMMC-7721 cells. Therefore, the HepG2 and SMMC-7721 cells were selected for studying.

### MTT and colony formation assay

For the MTT assay, HepG2-Vector and SMMC-7721-Vector, as well as HepG2-Kin17 and SMCC-7721-Kin17 hepatocellular carcinoma cells were plated into 96-well plates at a density of 1×10^4^ cells/well. The following steps were performed as described previously (12). Briefly, 120 μl dimethylsulfoxide (Kangwei Century Co., Ltd.) was added to each well and agitated for 15 min (H97-A, Solarbio Science & Technology Co., Ltd, Beijing, China). The absorbance was measured at a wavelength of 492nm. For the colony formation assay, the concentration of the cells was adjusted to 180 cells/dish and the cells were incubated at 37°C. The culture media was changed every 2 days. After two weeks, cells were fixed with methanol and stained with Trypan blue (Solarbio Science & Technology Co., Ltd.). The average number of colonies in five visual fields was counted under a microscope (YS100, Nikon, Tokyo, Japan).

### In vivo tumor growth assay

For the xenograft experiment, four- to six-week-old female nude mice (BALB/cA nu/nu) were obtained from the Animal Center of Xinxiang Medical University (Xinxiang, China). HepG2-Kin17 (n=4) and SMMC-7721-Kin17 cells were implanted subcutaneously into the back of the nude mice (BALB/cA nu/nu). Simultaneously, HepG2-Vector (n=4) and SMMC-7721-Vector cells were implanted under the same experimental conditions. Each group had four nude mice.

The maximum (a) and minimum (b) diameters of the tumors were measured, and tumor volume was calculated according to the following formula: Tumor volume = a × b^2^/2. Five weeks following tumor implantation, the nude mice were euthanized and the tumors were excised.

### Statistical analysis

Statistical analysis was performed using SPSS 13.0 (SPSS, Inc., Chicago, IL, USA). Comparisons between two groups were conducted by Student’s t-test. Results are expressed as the mean ± standard deviation. P<0.05 was considered to indicate a statistically significant difference.

## Results

### Kin17 expression in hepatocellular carcinoma tissues and hepatocellular carcinoma cell lines

**Figure 1:**
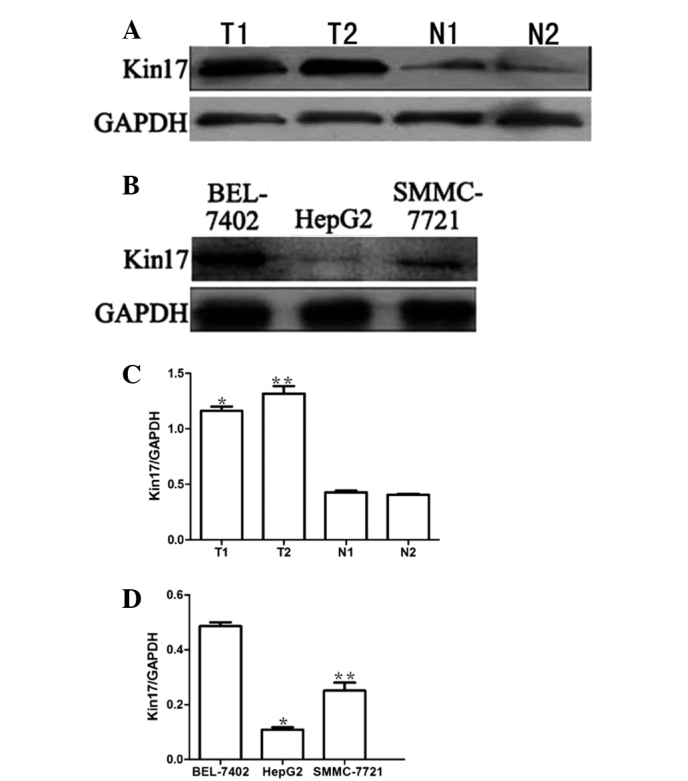
Kin17 expression in hepatocellular carcinoma tissues and cell lines. (A) Kin17 expression was increased in hepatocellular carcinoma tissues (1.347±0.29) compared with that in the corresponding normal tissues (0.394±0.13). (B) Kin17 expression was increased in BEL-7402 cells compared with that in HepG2 and SMMC-7721 cells. GAPDH was used as control. Two representative tissue samples were selected from the eight tissue pairs. (C) Kin17 expression was significantly higher in T1 and T2 compared with N1 and N2. *P<0.05 T1 vs. N1;^**^P<0.05 T2 vs. N2. (D) Kin17 expression levels were significantly lowe in the HepG2 and SMMC-7721 cells, compared with teh BEL-7420 cells. ^*^P<0.05, HepG2 vs. BEL-7420; **P<0.05, SMMC-7721 vs. BEL-7420) N1 and N2, normal tissue samples 1 and 2; T1 and T2, tumor tissue samples 1 and 2.

Kin17 expression was examined by western blotting in the human hepatocellular carcinoma and normal tissues, and in the HepG2, SMMC-7721 and BEL-7402 human hepatocellular carcinoma cell lines. As shown in Fig. 1, Kin17 expression was significantly higher in the hepatocellular carcinoma tissues (1.347±0.29) compared with that in the corresponding normal tissues (0.394±0.13) (P<0.05). Furthermore, the expression levels of Kin17 in the BEL-7402 cells were markedly higher than those in the HepG2 and SMMC-7721 cells (0.405±0.18 vs. 1.239±0.21 and 0.137±0.11, respectively) (P<0.05).

### Effects of Kin17 on cyclin D1 and p27Kip1 expression

**Figure 2:**
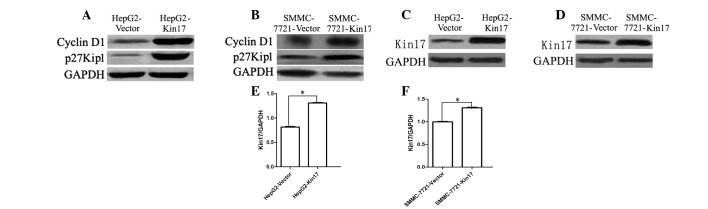
Expression of cyclin D1, p27KIP1 and Kin17 in HepG2 and SMMC-7721 cells. (A) Upregulation of Kin17 increased the expression of cyclin D1 and p27Kip1 in HepG2 cells. (B) Kin17 induced the expression of cyclin D1 and p27KIP1 in SMMC-7721 cells. (C) Kin17 expression was increased in HepG2 following transfection with Kin17 cDNA. (D) Kin17 staining was enhanced in SMMC-7721 cells following transfection with Kin17 cDNA. GAPDH was used as control. (E and F) Expression of Kin17 was significantly increased in HepG2-Kin17 and SMMC-7721-Kin17 cells compared with the control groups. ^*^P<0.05 vs. control group.

Western blot analysis revealed that the HepG2-Kin17 and SMMC-7721-Kin17 cells displayed increased expression levels of cyclin D1 and p27Kip1 protein compared with the HepG2-Vector and SMMC-7721-Vector cells (P<0.05; Fig. 2). These results demonstrated that the Kin17 might be, at least in part, associated with cyclin D1 and p27Kip1 expression.

### Upregulation of Kin17 expression promotes the proliferation of hepatocellular carcinoma cells in vitro

Taking into account the low endogenous expression of Kin17 in HepG2 and SMMC-7721 cells, these two cell lines were utilized to explore the role of Kin17 on the proliferation of hepatocellular carcinoma cells. As shown in Fig. 2, the expression of Kin17 was enhanced in HepG2 and SMMC-7721 cells following transfection with Kin17 cDNA.

**Figure 3:**
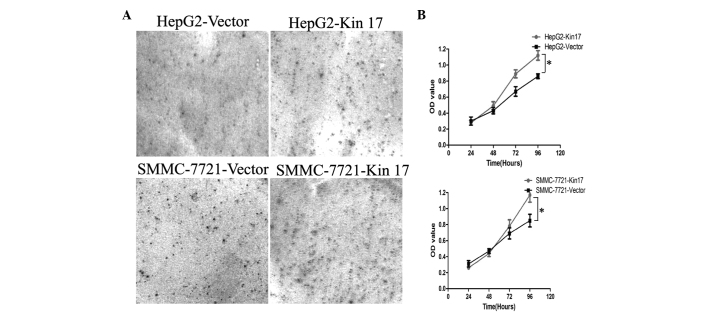
Kin17 overexpression promotes the growth of HepG2 and SMMC-7721 cells. (A) HepG2-Kin17 and SMMC-7721-Kin17 cells formed more and larger colonies than the relative control groups (magnification, ×100). (B) The MTT assay revealed that the overexpression of Kin17 enhanced the proliferation of HepG2 and SMMC-7721 cells. ^*^P<0.05.

The Colony formation assay demonstrated that the number of colonies formed by the HepG2-Kin17 group (367±21) was significantly higher than that of the HepG2-Vector group (189±13) (P<0.05; Fig. 3A). Similarly, the number of colonies formed by the SMMC-7721-Kin17 group (394±25) was greater than that of the SMMC-7721-Vector group (226±16) (P<0.05), indicating that the results were not specific to HepG2 cells. The MTT assay revealed that overexpression of Kin17 enhanced the proliferation of HepG2 and SMMC-7721 cells. Three days after seeding, the cell number of the HepG2-Kin17 group was significantly higher than that of the HepG2-Vector group (P<0.05) (Fig. 3B). Subsequently, the proliferation stimulating effect of Kin17 was affirmed in SMMC-7721 cells.

### Upregulation of Kin17 expression accelerated tumor growth in vivo

**Figure 4:**
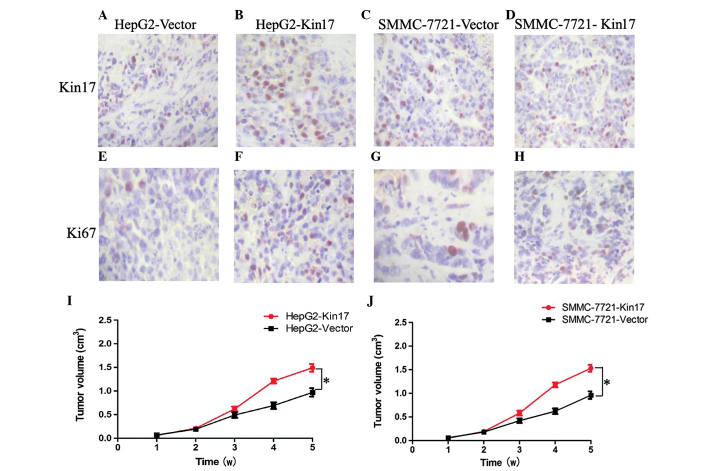
Expression of Kin17 and Ki-67 was upregulated in (B and F) HepG2-Kin17 and (D and H) SMMC-7721-Kin17 cells compared with the relative control groups (A and E; C and G) (magnification, ×200). Tumor volume in the (I) HepG2-Kin17 and (J) SMMC-7721-Kin17 groups was significantly larger than that of the relative control groups. ^*^P<0.05.

Compared with the HepG2-Vector (12.6±1.9)and SMMC-7721-Vector cells (18.8±2.3), Kin17 expression was increased in the mice transfected with HepG2-Kin17 (29.7±3.1) and SMMC-7721-Kin17 cells (32.5±4.1) (P<0.05) (Fig. 4A–D). By using Ki-67, it was identified that the proliferation index (PI) was significantly increased in the HepG2-Kin17 (20.3±1.8; Fig. 4F) and SMMC-7721-Kin17 (25.9±1.2; Fig. 4H) groups compared with the HepG2-Vector (8.2±0.9; Fig. 4E) and SMMC-7721-Vector (9.7±0.6; Fig. 4G) groups (P<0.05 for both). Furthermore, mice injected with HepG2-Kin17 and SMMC-7721-Kin17 cells had a larger tumor volume than that of animals receiving HepG2-Vector and SMMC-7721-Vector cells (P<0.05; Fig. 4I and J). Collectively, these results indicated that upregulation of Kin17 accelerated tumor growth *in vivo*.

## Discussion

The Kin17 protein is expressed in a wide variety of tissues and is mainly located in the nucleus (6,7,13,14). It is reported that Kin17 is involved in complex cellular processes, such as DNA replication and cellular response to DNA damage (15,16). Moreover, compared with normal human fibroblasts, elevated levels of Kin17 protein have been found in immortalized human fibroblasts (15–18). This finding suggested that Kin17 might be involved in the tumorigenesis of cancer.

Serum-stimulated mouse fibroblasts showed Kin17 mRNA expression increased significantly and promoted cell growth compared with the cells without serum stimulation (3). Similarly, Zeng *et al* also revealed overexpression of Kin17 promoted DNA replication and cell proliferation in breast cancer cells (18). In the present study, Kin17 was revealed to be overexpressed in hepatocellular carcinoma patient samples compared with the corresponding normal tissue samples. Moreover, upregulation of Kin17 expression promoted the growth of hepatocellular carcinoma cells *in vitro* and *in vivo*. At the cell level, Kin17 promoted cell proliferation and colony formation. In the hepatocellular carcinoma cell xenograft model, tumors of mice injected with HepG2-Kin17 and SMMC-7721-Kin17 cells were larger than those of mice implanted with HepG2-Vector and SMMC-7721-Vector cells. Moreover, the PI reflected by Ki-67 in the Kin17-overexpressing hepatocellular carcinoma cells was significantly higher than that in the control hepatocellular carcinoma cells. The association between enhanced levels of Kin17 and altered tumorigenic characteristics suggests that Kin17 is vital for the growth of hepatocellular carcinoma. Paradoxically, Kannouche *et al* reported that overproduction of Kin17 inhibited the proliferation of human epithelioid cervical carcinoma (HeLa) and non-small lung cancer cells (H1299) (19). Further study found that this was correlated with changes in nuclear morphology and with a decrease in DNA replication rate. The discrepancy in the results may be due to the different types of cancer studied.

The molecular mechanism by which Kin17 promotes cell proliferation is unknown. Cyclin D1 is one of the important regulators of G1/S transition (20), and p27Kip1 is one of the key regulators of cell cycle (21). In the current study, it was identified that Kin17 upregulated cyclin D1 expression and downregulated p27Kip1 expression. The results indicated that the effect of Kin17 in promoting the proliferation of hepatocellular carcinoma cells correlates with the expression of cyclin D1 and p27Kip1.

In conclusion, the present has study demonstrated that increased Kin17 expression promotes the growth of hepatocellular carcinoma *in vitro* and *in vivo*. However, further studies are required to clarify the exact mechanism of Kin17 in the tumorigenesis of hepatocellular carcinoma. The important role of Kin17 in the proliferation of cancer cells may present as a useful target for the treatment of hepatocellular carcinoma.
